# Relationships between the Pathogen *Erysiphe alphitoides*, the Phytophagous Mite *Schizotetranychus garmani* (Acari: Tetranychidae) and the Predatory Mite *Euseius finlandicus* (Acari: Phytoseiidae) in Oak

**DOI:** 10.3390/insects12110981

**Published:** 2021-10-29

**Authors:** Slobodan Milanović, Katarina Mladenović, Bojan Stojnić, Alejandro Solla, Ivan Milenković, Vanja Uremović, Ayco J. M. Tack

**Affiliations:** 1 Faculty of Forestry, University of Belgrade, Kneza Višeslava 1, 11030 Belgrade, Serbia; vanjauremovic@gmail.com; 2 Faculty of Forestry and Wood Technology, Mendel University in Brno, Zemědělská 3, 613 00 Brno, Czech Republic; 3 Institute of Forestry, Kneza Višeslava 3, 11030 Belgrade, Serbia; katarina.mladenovic@gmail.com; 4 Faculty of Agriculture, University of Belgrade, Nemanjina 6, 11080 Belgrade, Serbia; bstojnic@agrif.bg.ac.rs; 5 Faculty of Forestry, Universidad de Extremadura, Avenida Virgen del Puerto 2, 10600 Plasencia, Spain; asolla@unex.es; 6 Department of Ecology, Environment and Plant Sciences, Stockholm University, 106 91 Stockholm, Sweden; ayco.tack@su.se

**Keywords:** *Quercus robur* forest, epiphytic fungus, mites, multi-trophic interactions

## Abstract

**Simple Summary:**

Knowledge about the relationships between plant pathogens, arthropods, and their natural enemies is scarce. We studied the relationships between the plant fungal pathogen, *Erysiphe alphitoides*, the phytophagous mite *Schizotetranychus garmani*, and the predatory mite *Euseius finlandicus* in leaves of pedunculate oak. In June, July and August 2016, in 30 trees located in three forests near Belgrade, Serbia, the presence of *E. alphitoides*, *S. garmani* and *E. finlandicus* was assessed. The occurrence of *E. alphitoides* was high where the population of *S. garmani* was high. However, the presence of the leaf pathogen *E. alphitoides* was not related to the amount of the predatory mite *E. finlandicus*. The relationships between powdery mildew and the two mite species were stable across time and space, and the presence of one mite was not influenced by the presence of the other mite.

**Abstract:**

Food webs on forest trees include plant pathogens, arthropods, and their natural enemies. To increase the understanding of the impact of a plant pathogen on herbivore-natural enemy interactions, we studied the powdery mildew fungus *Erysiphe alphitoides*, the phytophagous mite *Schizotetranychus garmani*, and the predatory and mycophagous mite *Euseius finlandicus* in pedunculate oak (*Quercus robur*) leaves. In June, July and August of 2016, we assessed the severity of powdery mildew, mite population density and adult female mite size in 30 trees in three forests near Belgrade, Serbia. In August, the infection severity of *E. alphitoides* related positively to the population density of *S. garmani* and negatively to the body size of *S. garmani* females. Throughout the vegetative season, the infection severity of *E. alphitoides* related positively to the population density of *E. finlandicus* but not to its body size. The effect of *E. alphitoides* on the population density and adult size of *S. garmani* was not mediated by the population density of *E. finlandicus*, and *vice versa*. Interactions were consistent in all forests and varied with the summer month. Our findings indicate that *E. alphitoides* can influence the average body size and population densities of prey and predatory mites studied, irrespective of predator-prey relationships.

## 1. Introduction

Trees interact with a large diversity of arthropods and microorganisms [1,2,3]. The harmful effect of pathogens and herbivores on tree health is generally additive [4], and the occurrence of natural enemies of herbivores counteracts this effect [5]. Previous research reported complex interactions between plant pathogens and insect herbivores [6,7]. Plant pathogens can influence herbivore preference and performance, but may also change the relationship between herbivores and their natural enemies, influencing population densities. Interactions between herbivores and pathogens have been reported as positive, neutral or negative, and are frequently asymmetric [7,8,9]. Few studies have addressed the impact of plant pathogens on the relationship between insect herbivores and their natural enemies, or explored the direct effects of plant pathogens on herbivores and the indirect effects mediated by natural enemies, simultaneously.

Plant pathogens can affect the performance of herbivores [10,11] and their natural enemies [12] and influence herbivore-natural enemy interactions [13,14]. The effect of plant pathogens on the performance of insect herbivores can be due to direct feeding, resource competition and plant-mediated effects [15,16]. Plant pathogens can also affect natural enemies by changing the population density, morphology and behaviour of herbivores [16,17] and by altering a plant’s physical structure and volatile emissions [18,19,20], although these effects are less studied. Tripartite interactions between crops, microorganisms and herbivores have been widely studied in recent decades [21,22], and some of these interactions included trees [23,24,25,26,27]. Most studies focused on insect herbivores [28,29,30] and a few addressed mites [31], but no study has examined the interaction between leaf pathogens, phytophagous mites, and their natural enemies. 

Knowledge of plant-microbe-arthropod interactions is crucial for our understanding of natural systems, agriculture and forestry [22]. From a biological control perspective, we can learn how to use microbes to reduce plant attack by herbivores and determine whether certain microorganisms can enhance or limit the population densities of the natural enemies of arthropods, such as predators or parasitoids [32]. From this applied perspective, it is essential to determine whether insights gained from studies are applicable to other forests and times of year. If solutions are not applicable to large areas and different times of the season, it is necessary to identify the factors that shape spatial and temporal variations in effectiveness and globally assess the impact of microorganisms on the interactions between herbivores and their natural enemies across multiple locations and over time.

Our objective was to examine the effect of a plant pathogen on predator-prey interactions through an observational study in a forest tree species. We focused on the powdery mildew fungus *Erysiphe alphitoides* (Griffon and Maubl.) Braun and Takam, the phytophagous mite *Schizotetranychus garmani* Pritchard and Baker (Acari: Tetranychidae), the predatory mite *Euseius finlandicus* (Oudemans) (Acari: Phytoseiidae) and the pedunculate oak *Quercus robur* L. oak tree. Powdery mildew and the two mite species are common in natural forest ecosystems in Serbia [33,34,35]. We addressed the following questions: (i) do powdery mildew severity and predatory mite population density influence the population density and adult female size of the phytophagous mite? (ii) do powdery mildew severity and phytophagous mite population density influence the population density and adult female size of the predatory mite? and (iii) are the relationships between powdery mildew, prey mite and predatory mite stable across oak populations and time? We hypothesised that the size and population density of the phytophagous mite are negatively affected by powdery mildew through competition for resources and negatively affected by the predatory mite through predation. We alternatively hypothesised a positive and additive effect of powdery mildew on predatory mite size and population density because the predatory mite can use the fungus as supplementary food, i.e., by being mycophagous. Finally, we hypothesised that the relationships between powdery mildew and the two mite species are stable across time (month of summer) and space.

## 2. Materials and Methods

### 2.1. Study System 

**Figure 1:**
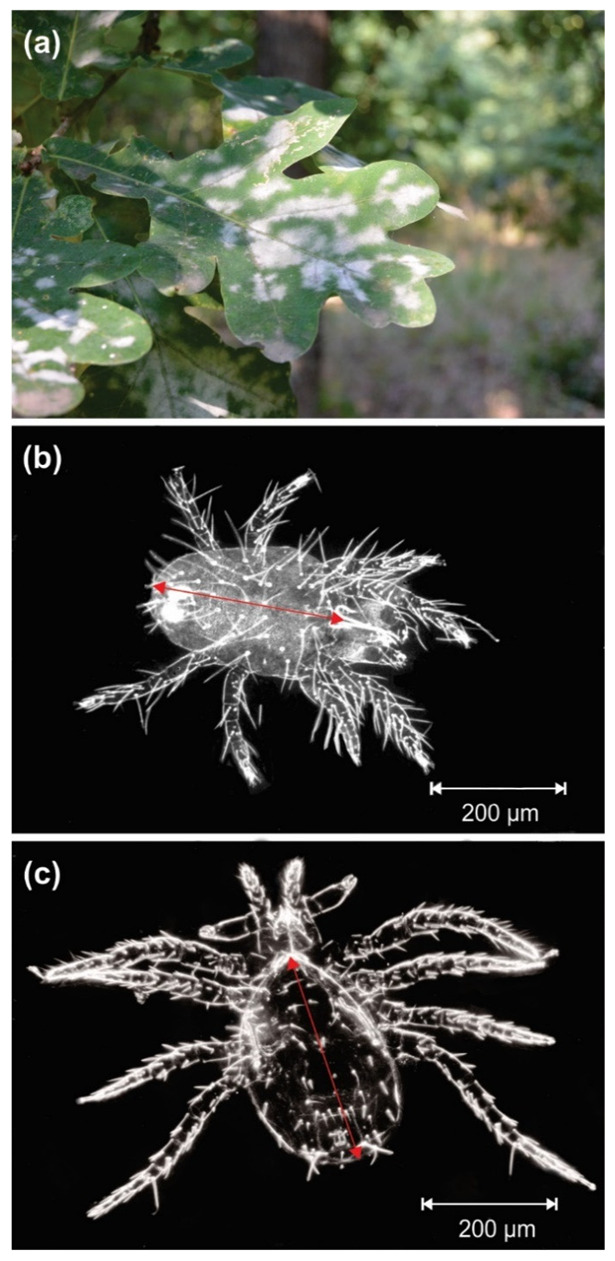
(**a**) White powdery patches of *Erysiphe alphitoides* on the upper side of a *Quercus robur* leaf, and dorsal side of adult females of (**b**) the phytophagous mite *Schizotetranychus garmani* and (**c**) the predatory mite *Euseius finlandicus. Red arrows* indicate idiosoma length.

Powdery mildew caused by *E. alphitoides* (Figure 1a), formerly known as *Microsphaera alphitoides*, is one of the major foliar diseases of oaks and had a significant role in oak decline in Europe [36,37]. Disease outbreaks caused by the winter moth (*Operopthera brumata* L.) and the tortrix moth (*Tortrix viridana* L.) are common in young leaves emerging after defoliation [38,39], and seedlings are more prone than old trees to infection [40]. Disease outbreaks can also affect mature trees if favourable environmental conditions for *E. alphitoides* occur in spring and summer, e.g., rain events, relative humidity of 76 to 96%, and temperatures around 20 °C [40], and when leaf flush synchronises with a high density of spores of the pathogen in the air [38]. Among the 20 oak species growing in Europe [41], the pedunculate oak is widespread and highly susceptible to *E. alphitoides* [40].

The family Tetranichydae (Acari) is one of the main groups of plant-feeding mite species, also known as spider mites [42]. Some spider mites are polyphagous [43], have a high developmental rate and fecundity and a short generation time, spread quickly across the landscape, and tend to develop resistance to pesticides rapidly [44]. As a consequence, spider mites can cause severe economic impact [45]. Worldwide, 117 species of the genus *Schizotetranychus* are recorded in angiosperm plants [43]. For most species of this genus, plant damage has not been documented or described, and only four species are categorised as at risk of damaging plants of economic importance [46]. As far as the authors know, *S. garmani* feeds exclusively on leaves.

Mites from the family Phytoseiidae are the most significant natural enemies of spider mites [47]. Phytoseiid mites can also feed on plant sap [48], and in some groups mycophagy has evolved as a supplement to predation [49]. The phytoseiid *Euseius finlandicus* (Figure 1c) is one of the most significant predators of phytophagous mites worldwide and can also feed on pollen, fungal spores and hyphae, eggs and larvae of insects, honeydew and plant liquids [50]. Trophic relationships between plants, fungi and mites can therefore be highly complex.

### 2.2. Experimental Procedure

**Figure 2:**
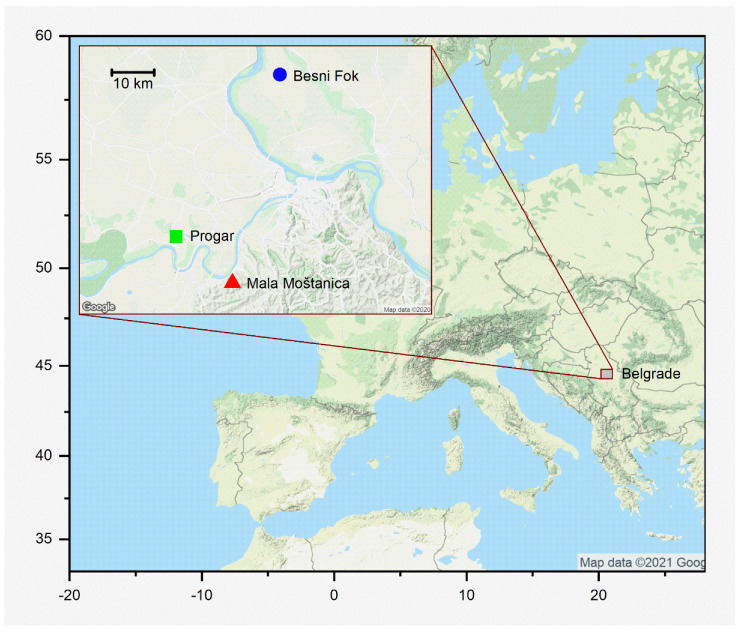
Location of the three *Quercus robur* forests studied, near Belgrade, Serbia.

To study the relationships between powdery mildew and phytophagous and predatory mites in oak, three *Q. robur* forests were selected in spring 2006. The forests were at Besni Fok (45.00156°, 20.40794°), Progar (44.7299°, 20.16236°) and Mala Moštanica (44.65258°, 20.29552°), near Belgrade, Serbia (Figure 2). At each site, 10 trees with powdery mildew but unaffected by any other disease were selected. Each tree was the experimental unit, and the study comprised 30 trees. Fifty mature leaves from each tree were sampled once a month, in June, July and August. In Belgrade in 2016, mean temperatures and total precipitation for each month were 22.5, 24.4 and 22.3 °C and 152, 35 and 61 mm, respectively (Republic Hydrometeorological Service of Serbia, www.hidmet.gov.rs/eng/osmotreni/naslovna.php, accessed on 25 October 2021). The 50 leaves per tree were collected at random from the lower parts of the canopies. All 1500 leaves sampled each month were carefully examined under a dissection microscope (Leica Wild M3Z, Leica Microsystems, Wetzlar, Germany) and their mites collected. Leaf area was estimated individually using SigmaScan Pro 5.0 software (Systat Software, Inc., San Jose, CA, USA). The mites were removed in a solution of ethanol and lactic acid [51], mounted in Hoyer’s medium [52] and identified with a phase-contrast microscope (Leica DMLS, Leica Microsystems, Germany) using specialised taxonomic keys of Tetranychidae [42,53,54,55,56] and Phytoseiidae [57,58,59,60] families. The population density of *S. garmani* and *E. finlandicus* was obtained in each tree by dividing the total number of mites of each species counted on 50 leaves by the total leaf area assessed in each tree. The length of the idiosoma of all the female individuals of *S. garmani* and *E. finlandicus* collected was measured. ‘Adult female size’, referring to idiosoma length (Figure 1b,c), was also averaged in each tree.

Ten of the 50 leaves sampled from each tree were placed separately into plastic Petri dishes. Leaf petioles were wrapped in cotton moistened with sterile distilled water. Powdery patches were observed under a binocular microscope (Olympus SZ-7, Tokyo, Japan) and fungal traits were observed in detail using a light Magnum T Trinocular microscope (CETI, Batley, UK). All traits were compared with those reported by Takamatsu et al. [61] and Braun et al. [62]. Based on the morphology of conidiophores and conidia, the number of asci in chasmothecia (i.e., sexual structures) and the shape of appendages, the powdery mildew observed was identified as *E. alphitoides*. In each tree, the percentage of leaf area affected by powdery mildew (infection severity) was estimated following Bert et al. [63]. Each of the 50 leaves sampled per tree was visually examined and assigned to one of the following damage groups: ‘0’ = no powdery mildew symptoms, ‘A’ = less than 50% of leaf surface with powdery mildew symptoms, ‘B’ = more than 50% of leaf surface with powdery mildew symptoms, and ‘C’ = whole leaf severely distorted and/or necrotic or dead. Whole tree infection severity was calculated using the equation of Bert et al. [63] as follows:

Severity = 0.25 × (percentage of leaves in group A) + 0.75 × (percentage of leaves in group B) + 1 × (percentage of leaves in group C)

Tree infection severity ranged from 0 to 100, corresponding to an infected leaf area percentage of 0 to 100%. We used this equation because the infection severity values followed a Gaussian distribution and to ensure our observations were comparable with those of previous studies [63].

### 2.3. Statistical Analysis

Statistical analyses were performed after obtaining ‘severity of *E. alphitoides*’, ‘population density of *S. garmani*’ and ‘population density of *E. finlandicus*’ in each tree. To assess the influence of powdery mildew and the predatory mite on the population density of the phytophagous mite, we used a linear mixed model (LMM). The model included ‘population density of *S. garmani*’ as the dependent variable, ‘forest’ and ‘month of summer’ as random and fixed factors, respectively, and ‘powdery mildew severity’ and ‘population density of *E. finlandicus*’ as covariates. To assess the influence of powdery mildew and the phytophagous mite on the population density of the predatory mite, a second LMM was used, including ‘population density of *E. finlandicus*’ as the dependent variable, ‘forest’ and ‘month of summer’ as random and fixed factors, respectively, and ‘powdery mildew severity’ and ‘population density of *S. garmani*’ as covariates. To assess the influence of powdery mildew and the predatory mite on adult female size of the phytophagous mite, a third LMM was used, including ‘adult female size of *S. garmani*’ as the dependent variable, ‘forest’ and ‘month of summer’ as random and fixed factors, respectively, and ‘powdery mildew severity’ and ‘population density of *E. finlandicus*’ as covariates. Finally, to assess the influence of powdery mildew and the phytophagous mite on the adult female size of the predatory mite, a fourth LMM was used, including ‘adult female size of *E. finlandicus*’ as the dependent variable, ‘forest’ and ‘month of summer’ as random and fixed factors, respectively, and ‘powdery mildew severity’ and ‘population density of *S. garmani*’ as covariates. To assess whether the relationships between powdery mildew and the mites were influenced by space (i.e., sampling locations) and time (i.e., time of the growing season), the models included the factors ‘forest’ and ‘month of summer’. Two-way interactions between variables were also included. Normality and homoscedasticity of the dependent variables were checked by KolmogorovSmirnoff and Bartlett’s tests. All analyses were performed with Statistica v.13 (TIBCO^®^ Software Inc., Palo Alto, CA, USA).

## 3. Results

**Figure 3:**
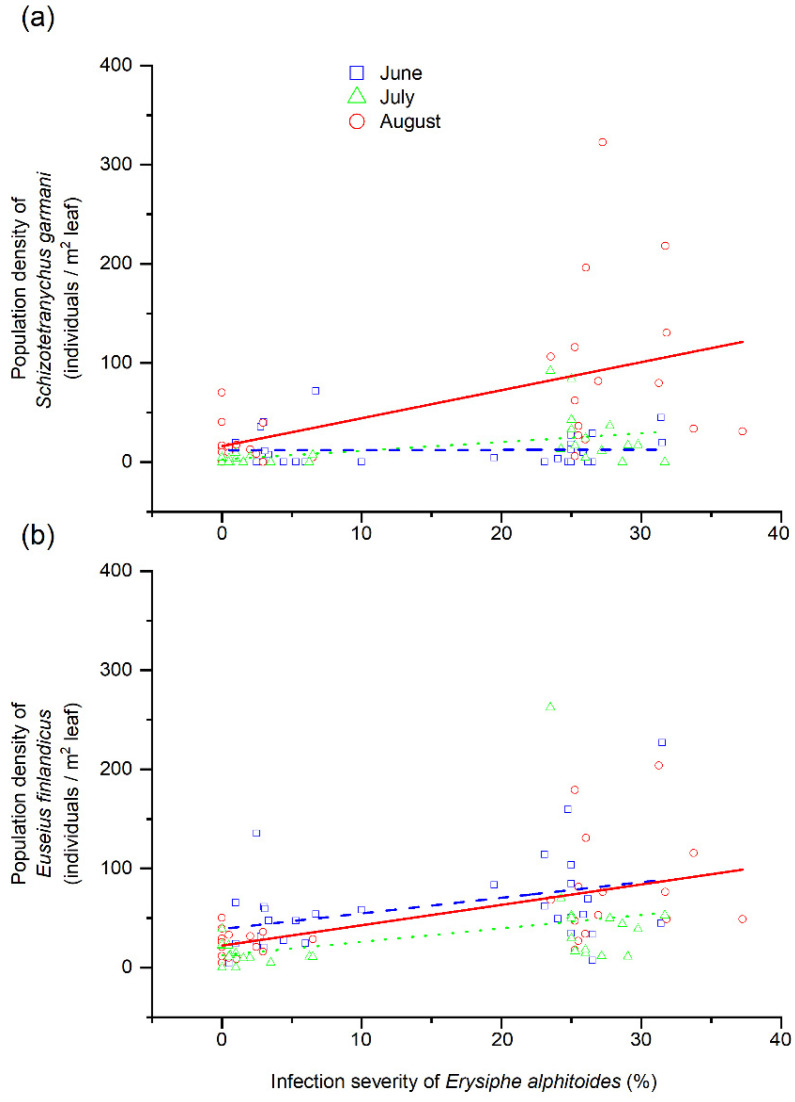
Relationships between severity of powdery mildew (*Erysiphe alphitoides)* in *Quercus robur* leaves and (**a**) population density of the phytophagous mite *Schizotetranychus garmani* or (**b**) population density of the predatory mite *Euseius finlandicus*. Severity ranged from 0 to 100, corresponding to an infected leaf area percentage of 0 to 100%. Circles, squares and triangles represent mean values at the tree level for June, July and August, respectively.

The population density of *S. garmani* significantly covaried with the severity of *E. alphitoides* (model 1 in Table 1). The positive relationship between these variables was conditioned by the month of summer (significant ‘month of summer’ × ‘severity of *E. alphitoides*’ in Table 1), and was non-significant in June and July (*p* > 0.05) and significant in August (*p* < 0.01; Figure 3a). The population density of *S. garmani* was not conditioned by the population density of *E. finlandicus* or by the forest (Table 1). The population density of *E. finlandicus* also positively covaried with the severity of *E. alphitoides* (model 2 in Table 1), although this relationship was not influenced by the month of summer (non-significant ‘month of summer’ × ‘severity of *E. alphitoides*’ in Table 1), and was significant in June, July and August (*p* < 0.05; Figure 3b). The population density of *S. garmani* was not conditioned by the population density of *E. finlandicus* or by the forest (Table 1).

**Figure 4:**
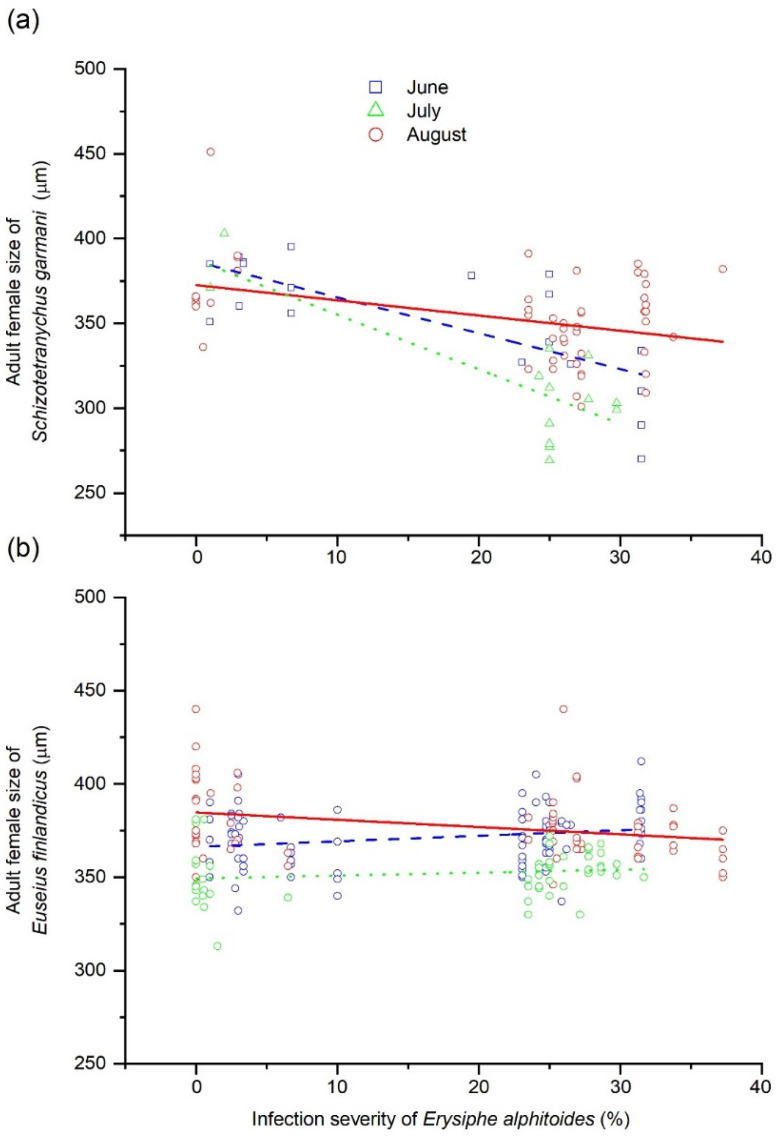
Relationships between severity of powdery mildew (*Erysiphe alphitoides)* in *Quercus* *robur* leaves and adult female idiosoma size of (**a**) the phytophagous mite *Schizotetranychus garmani* or (**b**) the predatory mite *Euseius finlandicus*. Severity ranged from 0 to 100, corresponding to an infected leaf area percentage of 0 to 100%. Circles, squares and triangles represent mean values at the tree level for June, July and August, respectively.

**Figure 5:**
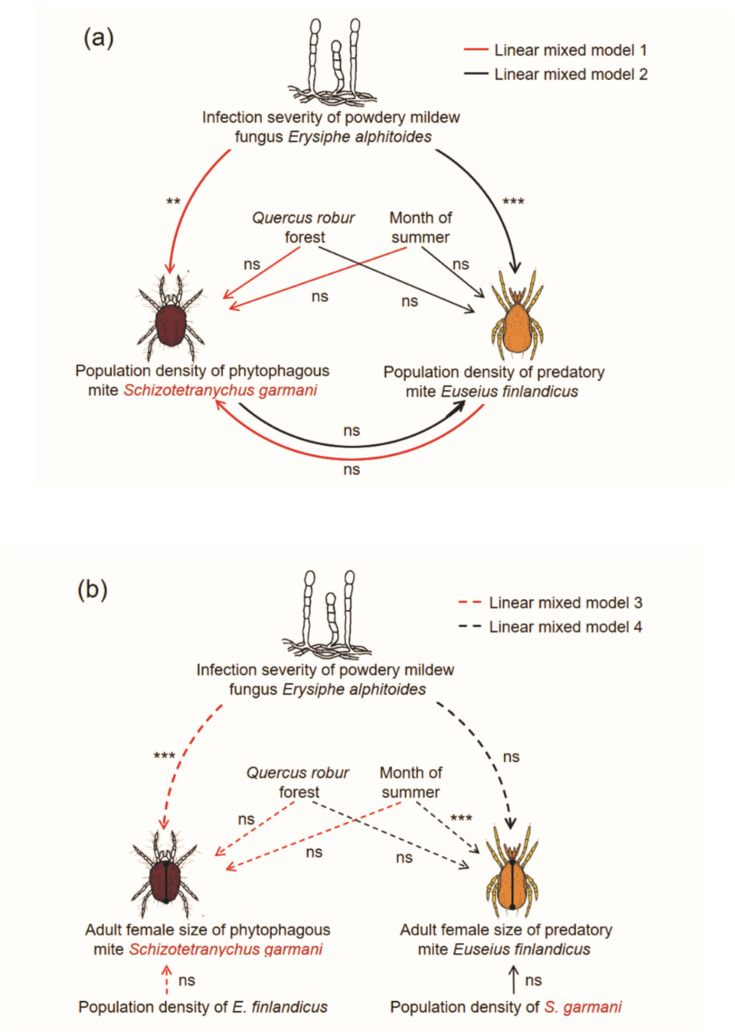
Summary of relationships in *Quercus robur* leaves between (**a**) population densities of *Schizotetranychus garmani* and *Euseius finlandicus* and (**b**) adult female idiosoma size of *S. garmani* and *E. finlandicus* and severity of *Erysiphe alphitoides*, the forest and the summer month. Asterisks indicate significances at *p* < 0.05 (**) and *p* < 0.01 (***), and no significance (ns), obtained from the models shown in Table 1.

The adult female size of *S. garmani* significantly covaried with the severity of *E. alphitoides* (model 3 in Table 1). The relationship was negative and conditioned by the month of summer (significant ‘month of summer’ × ‘severity of *E. alphitoides*’ in Table 1), and was significant in June and August (*p* < 0.05) and highly significant in August (*p* < 0.01; Figure 4a). The adult female size of *S. garmani* was not conditioned by the population density of *E. finlandicus* or by the forest (Table 1). Moreover, the adult female size of *S. garmani* was not affected by its population density (*r* = −0.13; *p* > 0.1). The adult female size of *E. finlandicus* was influenced by the month of summer only (model 4 in Table 1), and did not vary with its population density (*r* = −0.09; *p* > 0.1), between forests or during the summer (Figure 4b). The effects tested are summarised in Figure 5.

## 4. Discussion

The relationships between oak powdery mildew and phytophagous and predatory mites were studied during one vegetative season in three pedunculate oak forests. The levels of powdery mildew infection related positively to the population densities of mites and negatively to the adult female size of the phytophagous mite. In contrast to the influence of powdery mildew on mite population density, the two mite species did not interact with each other. Moreover, the effect of powdery mildew severity on the density of the phytophagous and predatory mites was not mediated by the density of the predatory and phytophagous mites, respectively. While the relationships between powdery mildew, the phytophagous mite and the predatory mite were stable across space, the relationship between powdery mildew and the phytophagous mite varied during summer.

The population density of the phytophagous *S. garmani* was positively influenced by the biotrophic foliar pathogen *E. alphitoides*. A positive relationship between the density of the spider mite *Tetranychus urticae* and the infection level of the powdery mildew *Podosphaera* spp. was observed on apples and sour cherries [64]. The population of a herbivore could be expected to decrease due to competition for resources with a pathogen [15], but previous research on oak indicated that interactions between *E. alphitoides* and insect species can range from negative to positive [13]. Mildew had a negative effect on the growth rate of the herbivore *Acronicta psi* L. and a positive effect on mite size and the parasitism of *Tischeria ekebladella* Bjerk. [13]. One explanation of why *S. garmani* abundance was positively related to the severity of *E. alphitoides* is that the phytophagous mite could have vectored the pathogen spores and, in turn, increased the level of infection [65], or could have facilitated penetration of the fungus into the host [66]. Our experiment was not manipulative but observational, thus it ignored if *E. alphitoides* made trees more susceptible to *S. garmani* or if *S. garmani* made trees more susceptible to *E. alphitoides*. Moreover, it ignored if a feedback loop consisting of increased susceptibility of trees to combined *E. alphitoides* and *S. garmani* stress occurred.

The decreased resource quality of leaves induced by pathogen infection [67] probably explains the smaller size of *S. garmani* in severely infected leaves. Inoculation of *Q. robur* seedlings with *E. alphitoides* was associated with the accumulation of secondary metabolites, such as phenols and lignins, in necrotic lesions and adjacent cells in infected oak leaves [68]. Herbivory by *Schizotetranychus baltazari* significantly altered the biochemical profile of curry (*Murraia koenigii* L.) leaves, leading to increased tannin content and decreased flavonoid and phenol, compared to non-infested plants [69]. Intraspecific competition and density dependence of food consumption probably do not explain why mites at the highest densities had small body sizes because the population densities observed were low. Food quality may also have impacted populations by changing the generation time of mites [70].

The severity of *E. alphitoides* also related positively to the population density of the predatory *E. finlandicus* mite, but not to its body size. No studies have examined the effect of plant pathogens on predatory mites. One study reported a higher population density of predatory spiders as a consequence of *Taphrina* sp. infection in *Populus trichocarpa* [25], in accordance with our findings of a positive effect of pathogen infection on the population of a predatory mite. One explanation for this positive effect is that *E. finlandicus* fed on the fungus. Several predatory mites, including *E. finlandicus* [50], are known to exhibit mycophagy [49]. Another explanation is that the biotrophic pathogen *E. alphitoides*, by inducing production of volatile compounds, such as methyl salicylate in *Q. robur* foliage [71], also attracted *E. finlandicus*, as occurs with other predatory mites [72].

Although many studies have investigated direct and plant-mediated interactions between fungal pathogens and arthropod herbivores [10,30,73], few have examined whether plant-pathogen-insect interactions are mediated by natural enemies [6]. In our study, the effect of powdery mildew severity on the population density and adult female size of *S. garmani* was not mediated by the population density of the predatory *E. finlandicus* mite. The absence of effects of *E. finlandicus* on its prey mite (and *vice versa*) was unexpected, because previous work in the laboratory had confirmed the trophic relationships between the mite species studied [74]. The strength of the predator-prey relationship may have been weakened by the presence of *E. alphitoides*.

The relationship between the severity of *E. alphitoides* and the population density and size of *S. garmani* varied significantly during the season. The predatory *E. finlandicus* was smallest in August. Few studies have tested plant-microbe-arthropod interactions by sampling multiple times during the growing season, and our data suggest patterns of variation in these interactions over time. According to the meteorological data series from Belgrade (not shown), 2016 was a typical meteorological year. In contrast to the observed seasonal changes of plant-fungus-mite interactions, the spatial location, i.e., the forest, was not influential. Our forests were far apart (Figure 2), and thus we tentatively conclude that the tree population does not influence the relationship between *E. alphitoides* severity and the population density (and female size) of mites in *Q. robur*. More years should have been included to assess landscape-level variation in the insect community structure, or to confirm the results summarised in Figure 5 in the long term. A study with more tree replicates would permit assessment of triple interactions. Moreover, the use of clonally replicated plant material intentionally placed in the forests would allow testing of the genotype effect.

## 5. Conclusions

Our findings indicate that severe leaf infection during summer of *Q. robur* by *E. alphitoides* was associated with increased population densities of the phytophagous *S. garmani* and the predatory (also mycophagous) *E. finlandicus* mites. Moreover, the population density of *E. finlandicus* did not influence the association between the pathogen and *S. garmani*, and the population density of *S. garmani* did not influence the association between the pathogen and *E. finlandicus*. Our findings also indicated that *E. alphitoides* can influence the average body size of the phytophagous *S. garmani* mite. The results contribute to our knowledge of natural food webs and plant-pathogen-arthropod interactions in forests.

## Figures and Tables

**Table 1:** Results of the linear mixed models for analysis of relationships and interactions between powdery mildew (*Erysiphe alphitoides*), a phytophagous mite (*Schizotetranychus garmani*) and a predatory mite (*Euseius finlandicus*) in leaves of *Quercus robur* from three forests, sampled for three months in summer. Significant *p*-values are indicated in bold.

Model	Dependent Variable	Predictor	Type	Degree of Freedom	*F* Ratio	*p*-Value
1	Population density of *Schizotetranychus garmani*	Forest (F)	Random effect	2	0.3	0.696
		Month of summer (M)	Fixed effect	2	0.2	0.750
		Severity of *Erysiphe alphitoides* (Ea)	Covariate	1	5.0	**0.027**
		Population density of *E. finlandicus* (Ef)	Covariate	1	0.9	0.331
		F × M	Random effect	4	2.0	0.104
		F × Ea	-	2	1.0	0.345
		M × Ea	-	2	6.1	**0.003**
		Ea × Ef	-	1	0.3	0.541
2	Population density of *Euseius finlandicus*	Forest (F)	Random effect	2	0.2	0.795
		Month of summer (M)	Fixed effect	2	1.0	0.353
		Severity of *Erysiphe alphitoides* (Ea)	Covariate	1	12.4	**<0.001**
		Population density of *S. garmani* (Sg)	Covariate	1	0.4	0.530
		F × M	Random effect	4	0.9	0.451
		F × Ea	-	2	0.7	0.470
		M × Ea	-	2	0.1	0.849
		Ea × Sg	-	1	0.1	0.738
3	Adult female size of *Schizotetranychus garmani*	Forest (F)	Random effect	2	2.9	0.079
		Month of summer (M)	Fixed effect	2	0.9	0.411
		Severity of *Erysiphe alphitoides* (Ea)	Covariate	1	9.3	**0.006**
		Population density of *E. finlandicus* (Ef)	Covariate	1	0.0	0.958
		F × M	Random effect	4	1.6	0.201
		F × Ea	-	2	1.6	0.214
		M × Ea	-	2	3.7	**0.044**
		Ea × Ef	-	1	0.0	0.865
4	Adult female size of *Euseius finlandicus*	Forest (F)	Random effect	2	0.4	0.620
		Month of summer (M)	Fixed effect	2	9.6	**<0.001**
		Severity of *Erysiphe alphitoides* (Ea)	Covariate	1	0.0	0.825
		Population density of *S. garmani* (Sg)	Covariate	1	0.4	0.507
		F × Ea	Random effect	4	0.6	0.627
		F × Ea	-	2	0.2	0.801
		M × Ea	-	2	1.5	0.222
		Ea × Sg	-	1	0.2	0.602

## Data Availability

Data may be requested from the corresponding authors.
